# Supplementary material for: Integration of visual and antennal mechanosensory feedback during head stabilization in hawkmoths
Source: eLife. 2022 Jun 27;11:e78410. doi: 10.7554/eLife.78410 (PMC9259029; doi:10.7554/eLife.78410)
Supplement: Supplementary file 1. [file elife-78410-supp1.docx]

Supplementary tables

Supplementary File 1a. List of Interquartile Range of Compensation error

| Groups | Twilight_2Hz | Twilight_6Hz | Dark_2Hz | Dark_ 6Hz |
| --- | --- | --- | --- | --- |
| *Flagella-intact* | 0.09 | 0.11 | 0.22 | 0.27 |
| *Flagella-clipped* | 0.06 | 0.13 | 0.03 | 0.07 |
| *Flagella-reattached* | 0.08 | 0.09 | 0.18 | 0.08 |
| *Sham* | 0.12 | 0.1 | 0.26 | 0.28 |
| *Johnston’s organ glued* | 0.05 | 0.22 | 0.18 | 0.18 |

Supplementary File 1b. List of interquartile ranges of Coherence

| Groups | Twilight_2Hz | Twilight_6Hz | Dark_2Hz | Dark_ 6Hz |
| --- | --- | --- | --- | --- |
| *Flagella-intact* | 0.00 | 0.02 | 0.05 | 0.12 |
| *Flagella-clipped* | 0.01 | 0.01 | 0.41 | 0.41 |
| *Flagella-reattached* | 0.00 | 0.01 | 0.05 | 0.09 |
| *Sham* | 0.01 | 0.02 | 0.04 | 0.14 |
| *Johnston’s organ glued* | 0.00 | 0.01 | 0.29 | 0.31 |

Supplementary File 1c. Details of the statistical tests used in various experiments in this paper

| **Groups compared** | **n size** | **Tests** | **p-value** | **Groups significantly different**  **(α = 0.05, post-hoc Nemenyi test)** |
| --- | --- | --- | --- | --- |
| **1. Twilight and dark compensation error**  (***Flagella-intact*, 2 Hz**) | Twilight: n = 8  Dark: n =8 | Wilcoxon  Signed-rank | 0.0078 | Not applicable |
| **2. Twilight and dark**  **compensation error**  (***Flagella-intact*, 6 Hz**) | Twilight: n = 8  Dark: n = 8 | Wilcoxon  Signed-rank | 0.5469 | Not applicable |
| **3. *Flagella-intact, Flagella-clipped and Flagella-reattached* compensation error** (**Twilight, 2 Hz**) | Flagella-intact: n=8, Flagella-clipped: n = 7, Flagella-reattached: n = 8 | Kruskal-Wallis test followed by Nemenyi test | 0.0107 | Flagella-intact, flagella-clipped |
| **4*. Flagella-intact, Flagella-clipped and Flagella-reattached* compensation error** (**Dark, 2 Hz)** | Flagella-intact: n=8, Flagella-clipped: n = 7, Flagella-reattached: n = 8 | Kruskal-Wallis test followed by Nemenyi test | 0.0133 | Flagella-intact, flagella-clipped |
| ***5. Flagella-intact, Flagella-clipped and Flagella-reattached* compensation error** (**Twilight, 6 Hz**) | Flagella-intact: n=8, Flagella-clipped: n = 7, Flagella-reattached: n = 8 | Kruskal-Wallis test followed by Nemenyi test | 0.0064 | Flagella-intact, flagella-clipped |
| ***6. Flagella-intact, Flagella-clipped and Flagella-reattached* compensation error** (**Dark, 6 Hz**) | Flagella-intact: n=8, Flagella-clipped: n = 7, Flagella-reattached: n = 8 | Kruskal-Wallis test followed by Nemenyi test | 0.0011 | 1. Flagella-intact, flagella-clipped  2. Flagella-clipped, flagella-reattached |
| **7.** ***Sham* and *Johnston’s organ glued* compensation error (Twilight, 2 Hz)** | Sham: n = 12  Johnston’s organ glued:  n = 7 | Wilcoxon  ranksum | 0.5358 | Not applicable |
| **8**. ***Sham* and *Johnston’s organ glued* compensation error (Dark, 2 Hz)** | Sham: n = 10  Johnston’s organ glued: n = 8 | Wilcoxon  ranksum | 0.0831 | Not applicable |
| 9. ***Sham* and *Johnston’s organ glued* compensation error (Twilight, 6 Hz)** | Sham: n = 10  Johnston’s organ glued: n = 7 | Wilcoxon  ranksum | 0.0097 | Not applicable |
| **10. *Sham* and *Johnston’s organ glued* compensation error (Dark, 6 Hz)** | Sham: n = 9  Johnston’s organ glued: n = 8 | Wilcoxon  ranksum | 0.0055 | Not applicable |
| **11. Flight bout duration of**  ***Control, sham and Head-restricted moths*** | Control: n = 11  Sham: n = 12  Head-restricted: n = 8 | Kruskal-Wallis test followed by Nemenyi test | 0.0001 | 1.Control, Head-restricted moths  2. Sham, Head-restricted moths |
| **12. Collision frequency of**  ***Control, sham and Head-restricted moths*** | Control: n = 11  Sham: n = 12  Head-restricted: n = 8 | Kruskal-Wallis test followed by Nemenyi test | 0.0002 | 1.Control, Head-restricted moths  2. Sham, Head-restricted moths |
